# Inhibition of Hedgehog-Signaling Driven Genes in Prostate Cancer Cells by *Sutherlandia frutescens* Extract

**DOI:** 10.1371/journal.pone.0145507

**Published:** 2015-12-28

**Authors:** Yuan Lu, Nicholas Starkey, Wei Lei, Jilong Li, Jianlin Cheng, William R. Folk, Dennis B. Lubahn

**Affiliations:** 1 Department of Biochemistry, University of Missouri, Columbia, MO 65211, United States of America; 2 MU Center for Botanical Interaction Studies, University of Missouri, Columbia, MO 65211, United States of America; 3 Division of Animal Science, University of Missouri, Columbia, MO 65211, United States of America; 4 Computer Science Department, University of Missouri, Columbia, MO 65211, United States of America; 5 Informatics Institute, University of Missouri, Columbia, MO 65211, United States of America; 6 Xiphophorus Genetic Stock Center, Texas State University, San Marcos, TX 78666, United States of America; University of Alabama at Birmingham, UNITED STATES

## Abstract

*Sutherlandia frutescens* (L) R. Br. (Sutherlandia) is a South African botanical that is traditionally used to treat a variety of health conditions, infections and diseases, including cancer. We hypothesized Sutherlandia might act through Gli/ Hedgehog (Hh)-signaling in prostate cancer cells and used RNA-Seq transcription profiling to profile gene expression in TRAMPC2 murine prostate cancer cells with or without Sutherlandia extracts. We found 50% of Hh-responsive genes can be repressed by Sutherlandia ethanol extract, including the canonical Hh-responsive genes *Gli1* and *Ptch1* as well as newly distinguished Hh-responsive genes *Hsd11b1* and *Penk*.

## Introduction

More than 200,000 new cases of prostate cancer (PCa) are diagnosed and almost 30,000 men die of this disease every year in the United States [1]. Prostate tumors are traditionally treated with hormonal antagonists, androgen ablation and/or chemotherapy. However, recurrence of prostate tumors is frequent and acquired resistance to traditional treatments is difficult to control. Novel approaches that can efficiently target and block the signaling pathways that lead to the recurrence, drug resistance and cancer progression are needed [2–4].

The abnormally activated Hedgehog (Hh) signaling pathway leads to advanced PCa and metastasis, and is important as well for other cancers such as medulloblastoma, basal cell carcinoma, small cell lung cancer, colorectal cancer and pancreatic cancer [2,3,5–7]. Blocking the activated Hh-signaling pathway in a prostate cancer xenograft model completely repressed growth of the aggressive PCa tumor. Inhibition of Hh-signaling pathway in other cancers has been shown to be an effective means to treat cancer. Vismodegib, a Hh-signaling inhibitor, has recently been approved by the US Food and Drug Administration (FDA) for basal cell carcinoma treatment [3,8–10].

Patched (Ptch) and Smoothened (Smo) are two important membrane proteins in the Hh-signaling pathway. Ptch represses Smo’s activity when it is not bound by its ligand Hh. When Hh ligand binds to Ptch, the inhibition is released and Smo is activated. Activated Smo leads to a signaling cascade whose downstream effects include the translocation and activation of Gli family of transcription factors and the transcription of pathway target genes, such as *gli1* and *ptch1*.

Blocking Hh-signaling using the plant secondary metabolite cyclopamine (or RNA interference of Gli) suppresses proliferation of several human prostate cancer cell lines [11]; however, cyclopamine’s potent inhibitory effect is not therapeutically favorable, because of its rapid clearance, non-specific toxicity and instability as well as off-target effects at high concentrations [3,12]. Therefore, identifying novel inhibitors of the Hh pathway is called for to inhibit prostate cancer proliferation and tumorigenesis.

*Sutherlandia frucescens* (*S*. *frucescens* or Sutherlandia) is a medicinal plant of South Africa that that is commonly known as “cancer bush” due to its application in cancer treatment [13–15]. Extracts of Sutherlandia have shown anti-proliferation and apoptotic effect in breast cancer cells, cervical cancer cells and Chinese Hamster Ovary cells [16–19]. However, the targets and mechanisms for these effects have not been identified. Previous studies in our laboratory revealed several botanical compounds that potentially prevent prostate cancer via Hh-signaling pathway inhibition [20,21], and we hypothesized that the anti-cancer effect of Sutherlandia is due to the inhibition of the Hh-signaling pathway. Using next-generation sequencing, we surveyed the Hh-signaling downstream gene expression alterations, as well as Sutherlandia extract responsive genes in the murine prostate cancer cell line TRAMPC2. When Sutherlandia extract was applied to TRAMPC2 cells with activated Hh-signaling, we observed repression of a large number of Hh-signaling target genes, indicating *S*. *frutescens* contains strong anti-Hh signaling compound(s). Consequently, *S*. *frutescens* maybe a potentially useful complementary treatment for advanced PCa and other Hh-signaling driven cancers.

## Materials and Methods

### Preparation of ethanol extract of *S*. *frutescens*

Ground *S*. *frutescens* powder was purchased from Big Tree Nutraceutical (Fish Hoek, South Africa) and characterized as described previously [22]. For preparation of ethanol extract of *S*. *frutescens*, 10g of ground *S*. *frutescens* leaf was mixed with 250 ml of 95% ethanol overnight. The supernatant was then harvested, centrifuged, sterilized, and stored at -80°C. Before use, *S*. *frutescens* ethanol extract [23] was dried down using speed-vacuum, and re-suspended into one-twentieth volume of DMSO with a final extract concentration of 84 mg/mL.

### Cell culture and reagents

Mouse TRAMPC2 prostate cancer cells were purchased from the American Type Culture Collection (ATCC) and cultured in complete RPMI 1640 medium (Life Technologies, Carlsbad, CA) supplemented with 10% Fetal Bovine Serum (FBS), insulin, and DHT.

Hh peptide Conditioned medium (Hh-CM) was generated from HEK293 cell line overexpressing Shh-N-terminal peptide (HEK293-ShhN, a kind gift from Dr. Phillip Beachy, Stanford University) [24]. HEK293-ShhN cells were grown to 80–90% confluence in DMEM medium containing 10% FBS, 1% Pen/Strep and 40 mg/mL G418. The medium was then replaced to DMEM containing 2% FBS, 1% Pen/Strep. After 24–30 h, the Hh-conditioned medium was collected and filtered through 0.22μm filters and stored at -80°C.

### Reverse Transcription and Real time PCR

Total RNA was isolated and purified from TRAMPC2 cells using RNeasy kit (Qiagen). 1000ng of total RNA was used to create cDNA libraries using Superscript III Reverse Transciptase (Invitrogen) with random primers and oligodT. Real time PCR was preformed using SYBR Green qPCR (iQ SUPERMIX, BioRad) on ABI7500 system. qPCR condition: 95 degree, 30 seconds; 60 degree, 40 seconds; 72 degree, 40 seconds. Primer sequences are listed in the S4 Table. Each qPCR assay was repeated 3 times on two biological replicates. T-test was performed on Hh-CM vs. control and Hh-CM+SFE vs. Hh-CM comparisons, p value cut-off is 0.05.

### RNA-Seq, Differentially Expressed Genes, and Bioinformatics Analysis

TRAMPC2 cells were treated with 8ug/ml and 80ug/ml SFE with or without Hh-CM for 24 hours, with each experiment repeated twice. Total RNA was extracted using RNeasy kit (Qiagen) and RNA concentration was quantified. The quality of total RNA was analyzed using Bioanalyzer (RIN score > 7.0). 2500ng total RNA from each of the two biological replicates of each experiment was used to generate sequencing libraries using TruSeq Stranded mRNA Sample Preparation kits (Illumina, CA). 50bp long single-end deep sequencing was performed by University of Missouri DNA Core using Illumina HiSeq 2000 system. The raw sequencing files, as well as the metadata, are submitted to NCBI GEO. GEO accession number: GSE75760. FASTX-Toolkit was used to remove the adaptor sequences, trim and filter low quality base call and low quality reads (http://hannonlab.cshl.edu/fastx_toolkit/). Filtered short sequencing reads were mapped to the murine genome (UCSC mm9) using TopHat2 and gene expression values were quantified using Subreads package FeatureCounts function [25–27]. Gene expression values were further used to calculate for library size and data set dispersion for differentially expressed gene analysis [28,29]. Briefly, the raw sequence reads of each gene were normalized to the library size of each sample and were converted to count per million (cpm). Gene differential expression was tested using R/Bioconductor package edgeR [30]. Differentially expressed genes are determined by Log_2_ Fold Change (Log_2_FC) and False Discovery Rate (FDR; Log_2_FC ≥1 or ≤-1; FDR≤0.05).

Gene set functional and pathway analysis were analyzed using Gene Ontology (GO) and KEGG pathway. The gene IDs of interest were converted to EntrezID and loaded to DAVID bioinformatics tools [31]. GO analysis and KEGG pathway analysis were then performed by setting all the GO terms and KEGG pathway genes as background genes. Overrepresented GO terms or pathways are determined by enrichment score (EASE≤0.1, gene count≥2).

## Results

### Sutherlandia altered gene expression

**Fig 1:**
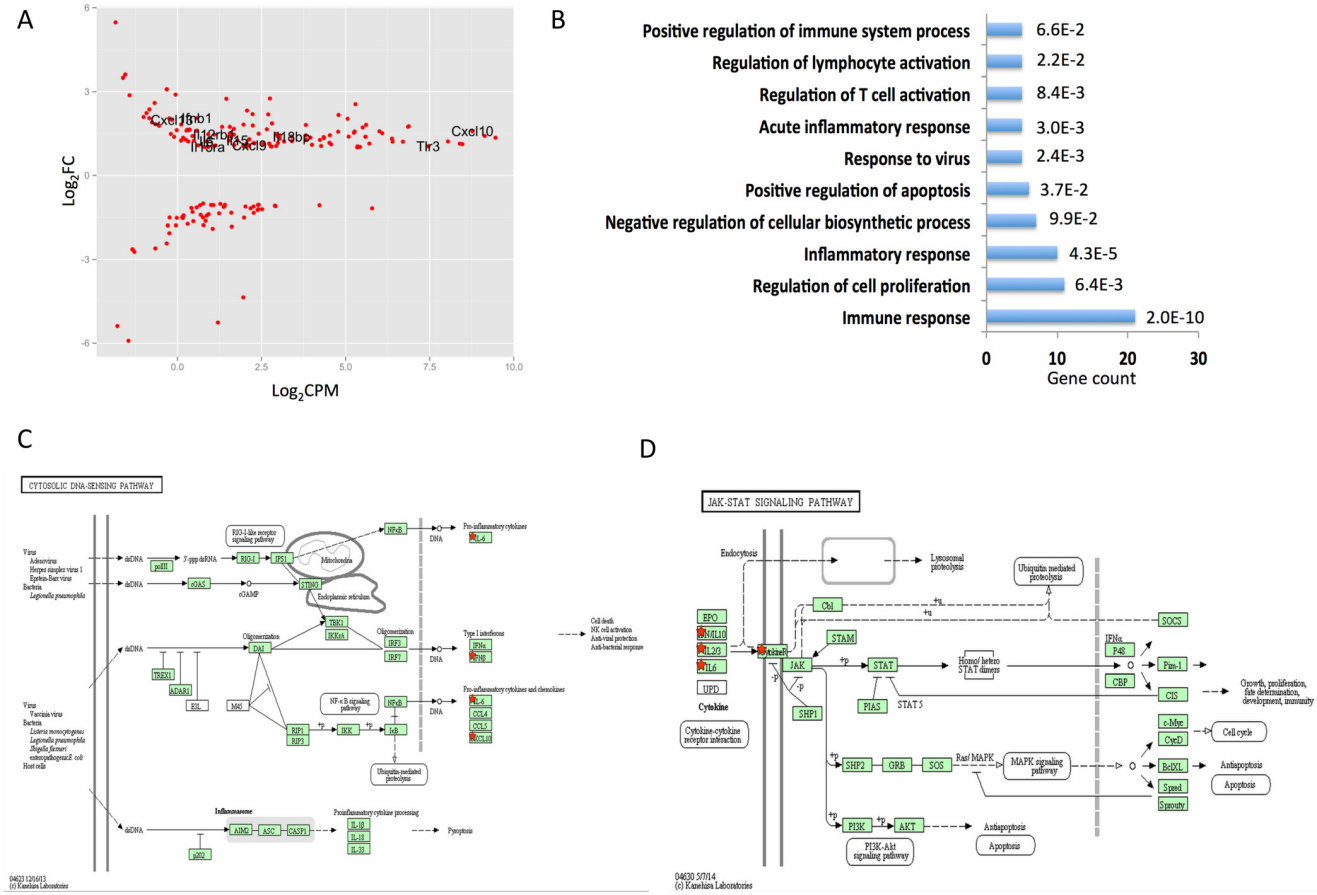
Sutherlandia Extract alters genes in TRAMPC2 cells. (A) Differentially expressed genes in response to Sutherlandia extract treatment. Genes that are related to (B, C, D) are labeled. (B) Gene Ontology analysis of Sutherlandia responsive genes. (C, D) KEGG pathway analysis of Sutherlandia responsive genes.

Sutherlandia ethanol extract (“SFE”) applied to cells at 8ug/ml did not result in any differentially expressed gene that met our statistical cutoff (data not shown). However, application of 80ug/ml SFE altered the mRNA expression of 204 genes, with 66 genes down-regulated, and 138 genes up-regulated (Fig 1A, S1 Table). Gene Ontology (GO) analysis and KEGG pathway analysis of these altered genes pointed to possible targets of SFE. The up-regulation of *Il6*, *Il15*, *Il18bp*, *cxcl9*, *cxcl10*, *cxcl13* and *tlr3* suggest targets to be immune and inflammatory related (Fig 1B) and pathway analysis underscored the NF-κB signaling pathway and the JAK-STAT signaling pathway: *Il6* and *cxcl10* being transcriptional targets of NF-κB and cytokines *Ifnb1*, *Il6*, *Il15* along with cytokines receptor *Il12rb1*, *Il15ra* being downstream of JAK-STAT signaling (Fig 1C and 1D).

### Sutherlandia represses Hedgehog-signaling pathway

**Fig 2:**
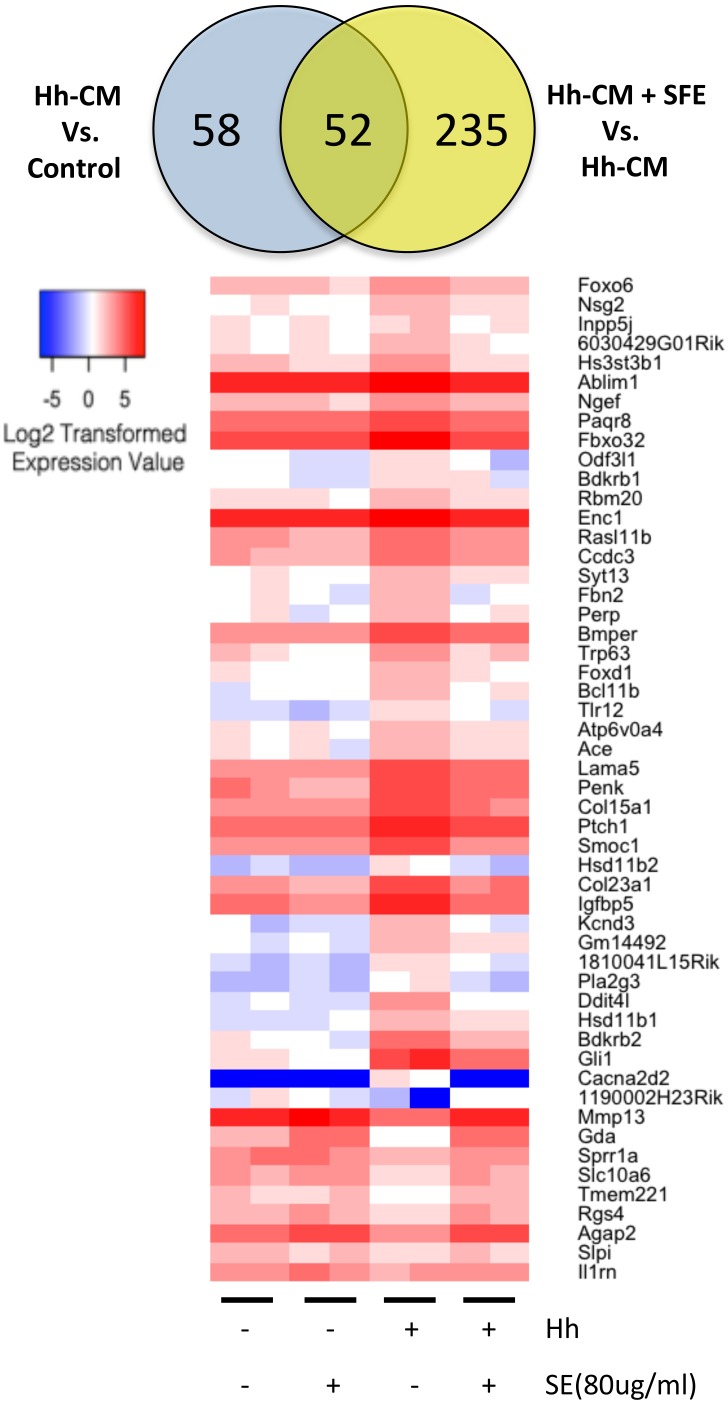
Heat map of Sutherlandia Extract altered Hedgehog-signaling pathway target genes expression. TRAMPC2 cell were treated with either Hh-CM or co-treated with Hh-CM and 80μg/ml SFE. Over 50% of Hh-responsive genes were repressed by SFE treatment. Gene expression values were represented by Log2 transformed normalized RNA-seq reads (Log2 count-per-million-reads) and color coded.

Hh ligand-enriched conditioned medium (Hh-CM) treatment of TRAMPC2 cells for 24 hours led to 110 differentially expressed genes, with 80 genes up-regulated and 30 genes down-regulated (S2 Table). Classical Hh-signaling target genes *gli1* and *ptch1* were up-regulated (Fig 2; S1 Fig) and indicate that TRAMPC2 cells are Hh-responsive.

**Fig 3:**
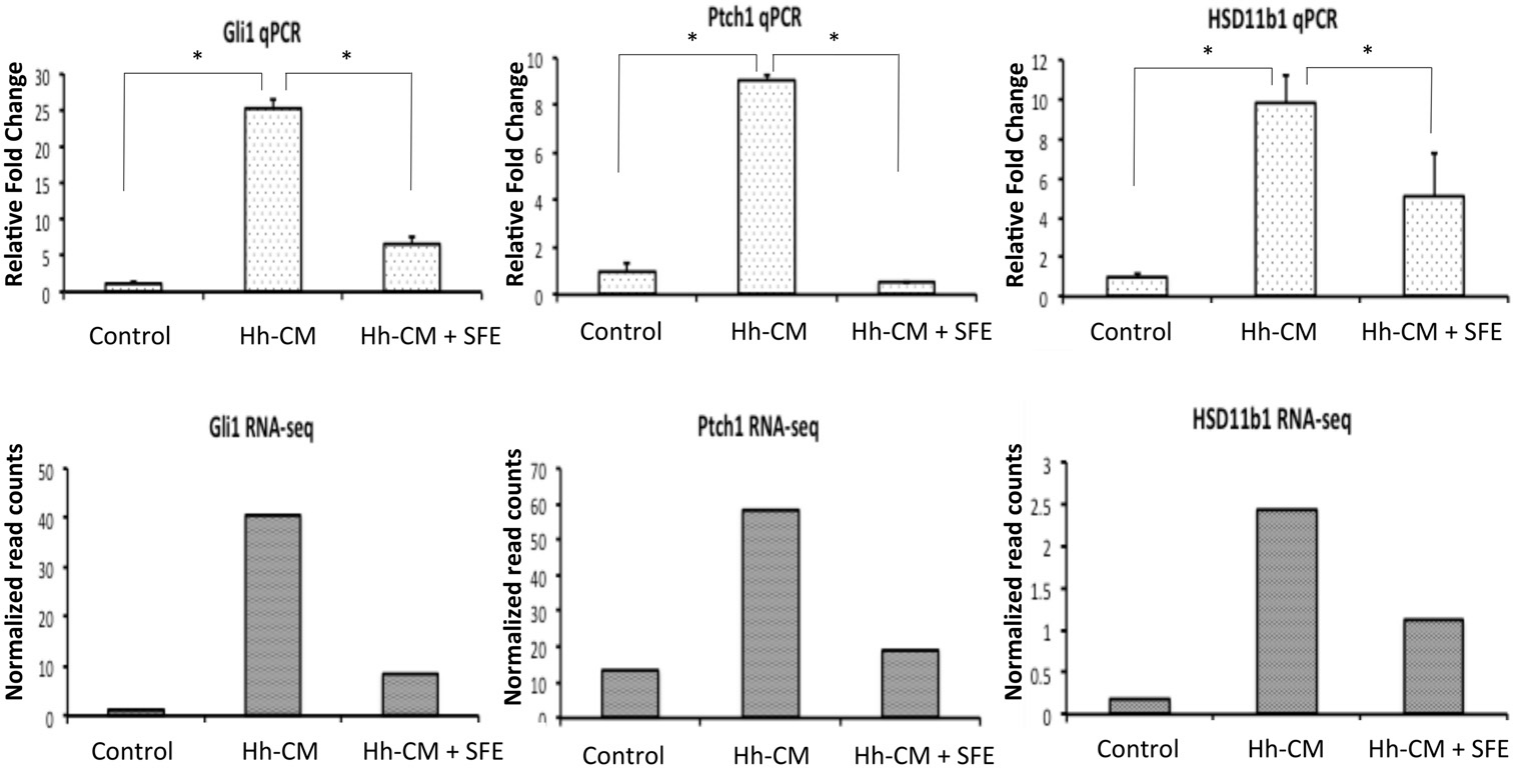
qPCR validation of RNA-seq result. Quantitative PCR for (A) *gli1*, (B) *ptch1*, and (C) *hsd11b1* was performed on Hh ligand treated and Hedgehog ligand and SFE co-treated TRAMPC2 cells. Transcripts concentrations were normalized to control. * indicates p<0.05. In the lower figure, transcripts concentrations of (A) *gli1*, (B) *ptch1*, and (C) *hsd11b1* are represented by quantified sequencing reads, in the form of counts-per-million-reads.

Co-treatment of SFE repressed 42 Hh-CM up-regulated genes, and stimulated 10 Hh-CM down-regulated genes (Fig 2). The Hh-signaling altered genes that can be repressed by SFE include the classical Hh-signaling target genes *gli1* and *ptch1*, as well as newly distinguished genes *hsd11b1* and *penk* (Fig 3; S1 Fig). Additional differentially expressed genes were immune and inflammatory related, including *Il6*, *Il15*, *cxcl9*, *cxcl10*, *cxcl11*, *ccl12*, *cxcl13*, *tlr3*, *tlr12*, *ifnb1* and *il12rb1* (S3 Table).

## Discussion

Sutherlandia, also known as cancer bush, is a prominent and widely used herbal medicine in South Africa [32]. Its targets and mechanisms of action toward cancer cells are largely unknown. In studies related to this, we have observed Sutherlandia extract to be growth inhibitory in several PCa cell lines and found the administration of Sutherlandia to TRAMP mice decreases the incidence of poorly differentiated carcinoma [21].

With the hypothesis that the cell proliferation inhibition of SFE is due to its potential Hh-signaling pathway inhibition, we identified a group of Hh-signaling responsive genes in TRAMPC2 cells, and found SFE was capable of repressing 50% of them. This suggests SFE has strong Hh-signaling inhibitory effects.

Interestingly, the fetal adrenal gland after maternal consumption of *Veratrum californicum*, which contains Hh-signaling inhibitor cyclopamine, synthesized much less cortisol, cortisone and corticosterone than the maternal adrenal, indicating corticosteroid biosynthesis is also strongly related to Hh-signaling activity [33]. The Hh-altered genes found in TRAMPC2 cells as well as Hh-altered genes in embryonic fibroblast cells and prostate cells, included the cortisol-cortisone conversion enzyme coding gene Hydroxysteroid Dehydrogenase 11 Beta 1(*hsd11b1*). *Hsd11b1* responded to Hh-signaling activation, while Sutherlandia extract treatment, or Smo inhibitor treatment, can repress the Hh-signaling pathway’s effect on this gene [34]. We conclude that *hsd11b1* is an Hh-signaling responsive gene and we propose that the decreased cortisol and cortisone concentrations in the adrenal of Cyclops fetus may result from *Veratrum californicum* grazing of mother sheep consuming Cyclopamine and inhibiting the Hh-signaling pathway.

*Hsd11b1* is present in PC3 and LnCaP human prostate cancer cells, and the HSD11B1’s 11-dehydrogenase activity is preserved while the 11-reductase activity is not [35,36], indicating HSD11B1 is important for maintaining the concentration of glucocorticoid in prostate and prostate cancer cells. This result strongly relates prostate cortisol/ cortisone concentration to the Hh-signaling pathway, suggesting that cortisol/ cortisone concentration alteration maybe an important factor for prostate cancer development and that anti-Hh treatment can reverse these detrimental changes.

Another new Hh-signaling responsive gene we found is Proenkephalin (*Penk*), a hormone ligand for the Opioid Growth Factor Receptor (Ogfr) and a negative regulator of cell proliferation and tissue organization. When Penk is synthesized and binds to nuclear located Ogfr, an intracellular signaling pathway downstream of Ogfr is initiated and eventually causes the cell to enter G0 phase. Penk is a prostate stroma marker and gene expression analysis showed that Penk concentration is lower in prostate cancer than in normal prostate [37]. It is surprising to find that Penk is expressed in TRAMPC2, while DU145 is *Penk* null from another gene expression profile from our lab, indicating TRAMPC2 cells have stromal features, potentially from Epithelial Mesenchymal Transition (EMT). More surprisingly, we found *Penk* is Hh-responsive gene and Sutherlandia extract treatment decreases either basal level or Hh-stimulated Penk transcript concentration.

Although the Sutherlandia ethanol extract showed repression of a large number of the Hh-response genes, we do not think that SFE contains Smo inhibitor(s) like cyclopamine, GDC0449 or DY131, otherwise it would repress all of the Hh responsive genes. We propose SFE contain one or more active components that alter the activity of downstream Hh-signaling pathway, which interact with other signaling pathways, or alternatively they act at the level of Gli transcription factor level in a promoter specific manner.

In addition to the discovery of Hh-signaling inhibition effect, we also found genes that are up-regulated by Sutherlandia treatment with or without Hh-signaling activation. GO analysis showed that the majority of these genes are immune response related, including Cfb, Cfh, Gbp2, Gbp4, Gbp5, Gbp10, Cxcl9, Cxcl10, Cxcl11, Cxcl13, IL6, IL18bp, Oasl1, Rsad2, Sp110, Tgtp1, Tgtp2, Tlr3, Tnfsf10 and Vnn1 (S1 and S3 Tables). While most of the up-regulated genes are shown to be Hh-signaling independent, revealed by the fold change similarity with or without Hh treatment, we do find 5 genes, Agap2, Aw112010, Gda, Cxcl11 and Npsr1, which are stimulated much more in response to Sutherlandia when Hh-signaling is activated, indicating that Hh-signaling facilitates these genes’ response to Sutherlandia.

In summary, our gene expression survey of Hh-signaling responsive genes and Sutherlandia responsive genes in mouse prostate cancer TRAMPC2 cells showed Sutherlandia extract has strong Hh-signaling inhibition effects as judged by the number and percentage of Hh responsive genes that can be repressed by Sutherlandia treatment. Our RNA-seq results suggest that Sutherlandia is a strong anti-Hh drug candidate with strong immune system boosting activities.

## Supporting Information

S1 Fig RNA-seq reads of Hsd11b1 and Penk. (PDF)

S1 Table 80ug/ml SFE responsive genes. (PDF)

S2 Table Hh responsive genes. (PDF)

S3 Table Differentially expressed genes between co-treatment of Hh and SFE, and Hh-CM treatment. (PDF)

S4 Table Primer sequences. (PDF)
