# Supplementary material for: Inhibition of Hedgehog-Signaling Driven Genes in Prostate Cancer Cells by Sutherlandia frutescens Extract
Source: PLoS One. 2015 Dec 28;10(12):e0145507. doi: 10.1371/journal.pone.0145507 (PMC4694108; doi:10.1371/journal.pone.0145507)
Supplement: S1 Fig — (PDF) [file pone.0145507.s001.pdf]

Supporting information file S1 Figure

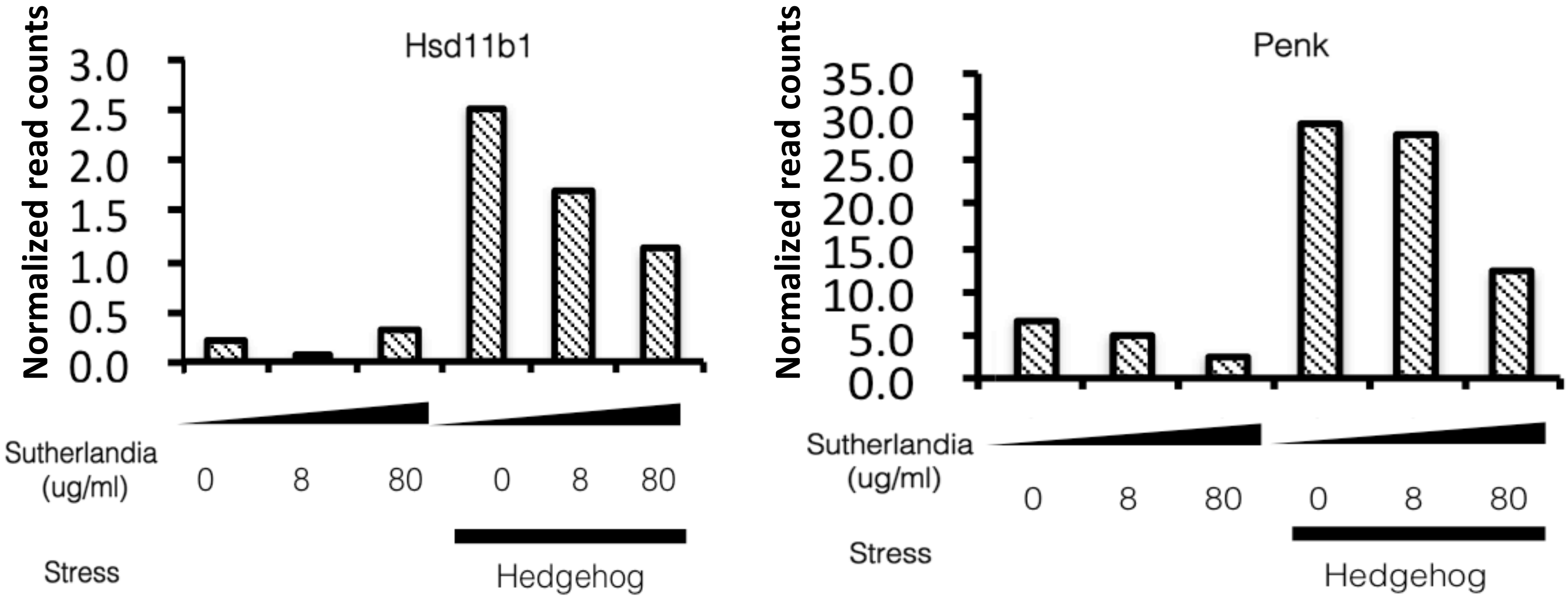

RNA-seq reads of Hsd11b1 and Penk Transcripts concentrations of (a) *hsd11b1*, (b) *penk* (c) *gli1* and (d) *ptch1* are represented by normalized deep sequencing reads, in the form of counts-per-million-reads (cpm). The cpm values are shown for the conditions with or without Hedgehog ligand.
