# Supplementary material for: Inhibition of Hedgehog-Signaling Driven Genes in Prostate Cancer Cells by Sutherlandia frutescens Extract
Source: PLoS One. 2015 Dec 28;10(12):e0145507. doi: 10.1371/journal.pone.0145507 (PMC4694108; doi:10.1371/journal.pone.0145507)
Supplement: S1 Table — (PDF) [file pone.0145507.s002.pdf]

Supplement Table 1. Sutherlandia Responisve Genes

GenBank Nucleotide Accession	Gene ID	symbol	logFC	logCPM	PValue	FDR
NM_177265	320802	330512M04Ri	-5.9146652	-1.4566541	7.17E-05	0.00254096
NM_139200	227929	Cytip	-5.3854713	-1.7873775	0.00209208	0.04178432
NR_002142	85029	Rpph1	-5.2618497	1.20309629	1.17E-07	7.76E-06
NR_001460	19782	Rmrp	-4.3601558	1.95692936	7.05E-07	4.14E-05
NM_001109897	22064	Trpc2	-2.728175	-1.280191	0.00180251	0.03741882
NM_172461	208583	Nek11	-2.6402217	-1.3367845	0.00263603	0.04965196
NM_001001454	57776	Ttyh1	-2.6396641	-1.3378074	0.00261047	0.04925541
NM_021324	57776	Ttyh1	-2.6396636	-1.3378074	0.00261133	0.04925541
NM_001109765	57776	Ttyh1	-2.6396635	-1.3378074	0.00261152	0.04925541
NR_037975	329763	Gm5105	-2.6395671	-1.3389897	0.00265803	0.04996704
NM_172854	241327	Olfml2a	-2.6064117	-0.6583565	0.00028436	0.00843928
NM_033037	12583	Cdo1	-2.4290148	-0.3214128	8.81E-06	0.00040068
NM_001243857	629059	Fam124a	-2.063395	-0.2412282	6.43E-05	0.00232171
NM_013599	17395	Mmp9	-1.9060814	1.04757274	5.59E-10	5.91E-08
NM_007955	13924	Ptprv	-1.8320641	1.6136538	3.18E-13	5.92E-11
NM_001082548	20733	Spint2	-1.7881928	-0.2876698	0.00044854	0.01210032
NM_011464	20733	Spint2	-1.7881919	-0.2876698	0.00044838	0.01210032
NM_028977	74511	Lrrc17	-1.7846061	-0.0457377	9.03E-05	0.00310227
NM_001166249	23945	Mgll	-1.7777247	0.77367911	3.14E-07	1.97E-05
NM_008438	16545	Kera	-1.7315281	0.30220246	4.23E-05	0.00160477
NM_025359	66109	Tspan13	-1.6354455	0.47077173	1.03E-05	0.0004654
NM_011844	23945	Mgll	-1.6201236	0.83705105	1.34E-06	7.46E-05
NM_001166251	23945	Mgll	-1.6201228	0.83705105	1.34E-06	7.46E-05
NM_011641	22061	Trp63	-1.5236325	0.16016847	0.00027916	0.00829346
NM_173749	210622	Pamr1	-1.5148706	-0.0224895	0.00104151	0.02423743
NM_009253	20717	Serpina3m	-1.507684	1.97912664	1.35E-11	1.86E-09
NM_008964	19217	Ptger2	-1.503752	-0.2337372	0.00153101	0.03298566
NM_028186	72293	Nkd2	-1.5026682	0.75702144	1.07E-05	0.00048154
NM_001127264	22061	Trp63	-1.4950798	0.14080784	0.00041774	0.01143226
NM_001127262	22061	Trp63	-1.4950797	0.14080784	0.00041775	0.01143226
NM_001190911	67703	Kirrel3	-1.436782	0.8680674	6.71E-06	0.00031695
NM_001190914	67703	Kirrel3	-1.4367819	0.8680674	6.70E-06	0.00031695
NM_026324	67703	Kirrel3	-1.4367818	0.8680674	6.69E-06	0.00031695
NM_001127259	22061	Trp63	-1.4318347	0.18064799	0.00049996	0.01327587
NM_001127260	22061	Trp63	-1.4318346	0.18064799	0.00050004	0.01327587
NM_177794	327766	Tmem26	-1.4078072	0.69024067	3.41E-05	0.00132319
NM_001190912	67703	Kirrel3	-1.3836785	0.92687195	8.04E-06	0.00036992
NM_020265	56811	Dkk2	-1.3557848	1.23705914	0.00020896	0.00649976
NM_001002927	18619	Penk	-1.3390075	2.26528107	1.22E-08	9.74E-07
NM_207668	56318	Acpp	-1.333823	1.60117397	3.06E-08	2.30E-06
NM_029407	75729	933432B09Ri	-1.3044217	0.4477175	0.00054928	0.01435982
NM_001163660	93960	Nkd1	-1.2405013	2.38002323	2.10E-10	2.39E-08
NM_027280	93960	Nkd1	-1.210898	2.40527052	6.41E-10	6.56E-08
NM_010865	17926	Myoc	-1.2106039	2.51942105	5.30E-11	6.68E-09
NM_009252	20716	Serpina3n	-1.1783063	5.78588177	3.15E-13	5.90E-11
NM_026820	68713	Ifitm1	-1.1768086	2.17167619	1.89E-09	1.77E-07
NM_001025581	268345	Kcnc2	-1.1628956	0.41310399	0.00149426	0.03243946
NM_018805	54710	Hs3st3b1	-1.1438385	0.56753708	0.00108098	0.02494006
NM_013560	15507	Hspb1	-1.1261849	1.37136918	6.27E-05	0.00227566
NR_033450	546546	Serpina3h	-1.1126857	2.02041073	1.17E-06	6.57E-05
NM_001112715	68713	Ifitm1	-1.1125768	2.2605837	7.87E-09	6.65E-07

NM_026629	68235	410066E13Ri	-1.110765	1.39503461	3.36E-05	0.00130493
NM_199240	214968	Sema6d	-1.0974877	2.91651827	2.04E-09	1.87E-07
NM_199241	214968	Sema6d	-1.0974875	2.91651827	2.04E-09	1.87E-07
NM_172537	214968	Sema6d	-1.0829407	2.89659515	2.01E-09	1.86E-07
NM_199238	214968	Sema6d	-1.0829405	2.89659515	2.01E-09	1.86E-07
NM_199239	214968	Sema6d	-1.0829402	2.89659515	2.00E-09	1.86E-07
NM_213616	381290	Atp2b4	-1.0676517	4.22225946	2.71E-17	9.17E-15
NR_038184	72575	430049B03Ri	-1.066709	0.66986152	0.00198327	0.04023217
NM_001099636	434128	Pnmal2	-1.0593591	1.07017643	0.00021094	0.00655421
NR_033210	216152	BC005764	-1.0588099	2.39275029	6.97E-06	0.0003281
NM_009131	20256	Clec11a	-1.0579008	0.9991129	0.00039765	0.01094536
NM_015755	26559	Hunk	-1.0495584	1.40890157	3.41E-05	0.00132306
NM_181681	216152	BC005764	-1.0475091	2.41351578	6.60E-06	0.00031354
NM_172874	242608	Podn	-1.0186213	1.27397566	0.00013461	0.0043783
NM_153417	225997	Trpm6	-1.0035416	0.76331278	0.00161094	0.0343438
NM_001081024	239122	Setdb2	1.00677704	5.34385584	1.49E-24	1.06E-21
NM_008358	16169	Il15ra	1.01509426	0.90627448	0.00076415	0.01867946
NM_001113514	104099	Itga9	1.01776811	0.80423259	0.00112664	0.02567272
NM_001034859	225594	Gm4841	1.02230601	5.42586739	7.73E-28	7.10E-25
NM_026271	67606	Fibin	1.02784356	2.05885364	4.30E-07	2.65E-05
NM_008161	14778	Gpx3	1.03777654	2.79845376	2.80E-09	2.52E-07
NM_001083929	14778	Gpx3	1.03777695	2.79845376	2.80E-09	2.52E-07
NM_012057	27056	Irf5	1.04442901	5.35243013	2.47E-28	2.51E-25
NM_126166	142980	Tlr3	1.04637626	7.45804052	2.72E-34	4.07E-31
NM_001039223	623781	Gm14137	1.04716001	2.77348869	1.99E-06	0.00010746
NM_020594	57432	Zc3h8	1.05670413	1.08749395	0.00014624	0.00470841
NM_001045540	620913	Gm12185	1.05685999	2.93442829	7.58E-12	1.08E-09
NM_011193	19200	Pstpip1	1.05695938	1.95424117	8.67E-07	4.96E-05
NM_001111267	211329	Ncoa7	1.05795429	4.27188945	1.61E-21	8.06E-19
NR_003967	209380	Gm4759	1.07303423	1.13039101	0.0005702	0.0147397
NM_008599	17329	Cxcl9	1.0751062	2.13625735	1.03E-07	6.98E-06
NM_008580	26408	Map3k5	1.07872158	0.61320048	0.00124339	0.02775541
NM_010426	15227	Foxf1a	1.08635404	1.68531487	2.00E-05	0.00082004
NM_031159	11810	Apobec1	1.09901575	3.99857434	3.11E-21	1.47E-18
NM_001134391	11810	Apobec1	1.09901575	3.99857434	3.06E-21	1.47E-18
NM_011704	22361	Vnn1	1.11549008	4.56883495	1.08E-07	7.26E-06
NM_010260	14469	Gbp2	1.12408264	8.47117711	1.78E-30	2.11E-27
NM_018738	16145	Igtp	1.13813281	8.39378127	2.71E-24	1.67E-21
NM_010430	15248	Hic1	1.13938581	2.72650582	4.81E-10	5.14E-08
NM_001033207	434341	Nlrc5	1.14205963	5.70411098	3.83E-28	3.63E-25
NM_175118	67446	Dusp28	1.14344432	2.54810711	2.11E-10	2.40E-08
NM_001098203	15248	Hic1	1.14464464	2.73005471	3.08E-10	3.45E-08
NM_007865	13388	Dll1	1.14996045	2.04848476	6.20E-08	4.41E-06
NM_001110517	667373	Gm14446	1.15945755	2.28858772	6.64E-08	4.62E-06
NM_001101605	667373	Gm14446	1.16537538	2.26545657	5.37E-08	3.86E-06
NM_028474	627626	110082D06Ri	1.16660335	2.98695209	2.16E-11	2.79E-09
NM_001033263	216439	Agap2	1.1720123	4.51790221	2.68E-24	1.67E-21
NM_008330	15953	Ifi47	1.2111851	6.70215049	8.29E-26	7.37E-23
NM_153564	229898	Gbp5	1.21322525	6.39440786	4.43E-43	1.15E-39
NM_148927	69217	Plekha4	1.21711307	3.16894405	1.28E-12	2.15E-10
NM_020557	22169	Cmpk2	1.21823336	8.04223495	5.37E-32	6.94E-29
NM_009641	11602	Angpt4	1.21940268	0.32000275	0.00199719	0.04044141
NM_031168	16193	Il6	1.22091392	0.86014699	7.11E-05	0.00253607

NM_001163621	71939	Apol6	1.22961613	3.40325989	4.61E-19	1.90E-16
NM_008791	18546	Pcp4	1.24489985	0.48972674	0.0002873	0.00850749
NM_183201	327978	Slfn5	1.24972626	1.53162005	7.26E-07	4.21E-05
NM_139065	245945	Rbm47	1.25060435	0.10646435	0.00206523	0.04143127
NM_001127382	245945	Rbm47	1.25060744	0.10646435	0.0020616	0.04140809
NM_008357	16168	Il15	1.25541106	1.78042767	2.59E-07	1.64E-05
NM_175397	109032	Sp110	1.2721794	4.23545725	2.73E-21	1.34E-18
NM_029000	74558	Gvin1	1.27766062	3.82602752	1.65E-24	1.08E-21
NM_001243040	100042856	Gm4070	1.27766063	3.82602752	1.66E-24	1.08E-21
NM_001243039	100042856	Gm4070	1.27766063	3.82602752	1.67E-24	1.08E-21
NM_001039160	74558	Gvin1	1.27766064	3.82602752	1.67E-24	1.08E-21
NM_007870	13421	Dnase1l3	1.28113532	0.24553465	0.00100207	0.02357023
NM_001142706	14962	Cfb	1.28431412	2.12160828	1.39E-08	1.09E-06
NM_008198	14962	Cfb	1.30043495	2.13263967	1.20E-08	9.66E-07
NM_011979	26464	Vnn3	1.31506368	3.03788115	9.56E-09	7.94E-07
NM_001204910	226691	AI607873	1.32091407	6.37618128	4.86E-30	5.53E-27
NM_028010	71939	Apol6	1.34041985	4.35394202	3.23E-32	4.38E-29
NM_178446	245945	Rbm47	1.35208693	0.17034245	0.00077988	0.01900641
NM_021384	58185	Rsad2	1.3534371	9.45368626	1.70E-37	3.03E-34
NM_177981	15114	Hap1	1.35925179	3.95444567	2.16E-20	9.77E-18
NM_001114679	667214	)30111J21Ril	1.37530804	4.34780422	8.10E-29	8.87E-26
NM_033079	110958	D6Mm5e	1.38236706	-0.0986012	0.00165268	0.03499765
NM_008880	18828	Plscr2	1.38645727	5.58642844	3.88E-40	8.50E-37
NM_001195084	18828	Plscr2	1.38732125	5.59310517	7.89E-40	1.60E-36
NM_010531	16068	Il18bp	1.39170674	3.39739945	4.29E-15	1.02E-12
NM_021893	60533	Cd274	1.39224884	5.08927762	6.57E-44	1.87E-40
NM_017466	54199	Ccrl2	1.40766298	2.97990137	1.52E-17	5.41E-15
NM_001164627	73167	Arhgap8	1.41076763	1.54285709	8.13E-09	6.83E-07
NM_001164628	73167	Arhgap8	1.4107679	1.54285709	8.13E-09	6.83E-07
NM_001205334	73167	Arhgap8	1.41076852	1.54285709	8.16E-09	6.83E-07
NM_028455	73167	Arhgap8	1.41077008	1.54285709	8.21E-09	6.85E-07
NM_008245	15242	Hhex	1.41340001	0.44554687	9.79E-05	0.00332507
NM_008332	15958	Ifit2	1.41762113	9.13520617	1.21E-54	6.89E-51
NM_172603	219131	Phf11	1.43704788	3.04405332	7.29E-14	1.49E-11
NM_010404	15114	Hap1	1.43810406	3.81820518	3.82E-18	1.41E-15
NM_025658	66607	Ms4a4d	1.44061335	4.53101867	8.45E-21	3.88E-18
NM_030218	78906	130017N09Ri	1.46339447	1.6780713	3.96E-10	4.33E-08
NM_008353	16161	Il12rb1	1.46524713	1.0961167	3.24E-07	2.02E-05
NM_001243837	109828	C7	1.47875497	0.91417922	1.29E-06	7.18E-05
NM_001039646	626578	Gbp10	1.48875383	2.92817199	3.11E-16	9.04E-14
NM_001037925	625360	BC147527	1.4890475	-0.1965436	0.00189439	0.03884447
NM_001039647	634650	Gbp11	1.49692296	2.41340368	1.85E-15	4.70E-13
NM_011243	218772	Rarb	1.51146519	1.55969777	1.07E-09	1.04E-07
NM_001135115	631323	Gm12250	1.51391773	6.08735771	2.33E-25	1.90E-22
NM_011303	20148	Dhrs3	1.53146574	5.24406222	7.73E-43	1.83E-39
NM_001172424	20148	Dhrs3	1.56756355	4.77651195	2.16E-37	3.62E-34
NM_001145164	100039796	Tgtp2	1.57669878	5.26751844	3.12E-28	3.07E-25
NM_145209	231655	Oasl1	1.58379242	6.00378894	1.99E-34	3.15E-31
NM_021274	15945	Cxcl10	1.58685635	8.77168188	1.08E-49	3.84E-46
NM_010680	16774	Lama3	1.60855485	0.57684575	5.18E-06	0.00025428
NM_001177351	107350	AW112010	1.60976051	0.26833941	3.84E-05	0.00147207
NM_009693	238055	Apob	1.62019098	0.34109446	1.67E-05	0.00070125
NM_011226	19331	Rab19	1.62454776	-0.0230984	0.00011631	0.00384029

NM_001004762	232889	Pla2g4c	1.63758405	0.28963638	2.57E-05	0.00103031
NM_001168504	232889	Pla2g4c	1.6375841	0.28963638	2.57E-05	0.00103031
NM_010999	18356	Olfr56	1.64749103	0.36155281	1.19E-05	0.0005254
NM_008392	16365	Irg1	1.65624784	2.65786322	1.06E-14	2.41E-12
NM_007431	11647	Alpl	1.67143914	1.67776427	3.90E-12	5.72E-10
NM_024237	70370	Fbln7	1.72175114	5.50557809	1.83E-39	3.47E-36
NM_019494	56066	Cxcl11	1.74124242	1.5567954	3.20E-12	4.74E-10
NR_038116	56066	Cxcl11	1.74124289	1.5567954	3.19E-12	4.74E-10
NM_001004174	433470	AA467197	1.7425282	1.32694665	1.51E-10	1.75E-08
NR_003520	17857	Mx1	1.74591369	6.85475477	8.18E-51	3.33E-47
NM_010846	17857	Mx1	1.76277061	6.87688233	7.38E-51	3.33E-47
NM_027990	71897	Lypd6b	1.78026738	-0.5452959	0.00113585	0.02579991
NM_001162938	100033459	Pydc3	1.79081605	2.23893655	1.32E-16	4.23E-14
NM_008620	17472	Gbp4	1.80962125	3.81761138	4.31E-31	5.34E-28
NM_009425	22035	Tnfsf10	1.81317667	5.60881664	2.91E-64	2.76E-60
NM_009888	12628	Cfh	1.82241835	1.96504909	1.27E-15	3.32E-13
NM_011861	23969	Pacsin1	1.82526506	0.11163244	9.11E-06	0.0004133
NM_028799	74176	Tgm5	1.83615307	-0.6497006	0.00108361	0.02494006
NM_001177349	623121	Pydc4	1.86584653	2.81132663	3.12E-23	1.82E-20
NM_001177350	623121	Pydc4	1.86584716	2.81132663	3.14E-23	1.82E-20
NM_009763	12182	Bst1	1.90269593	-0.7576323	0.0017679	0.03686163
NM_018866	55985	Cxcl13	1.99006289	-0.1634002	3.70E-05	0.00142251
NM_172648	226695	Ifi205	2.03196954	5.05860757	5.45E-82	7.75E-78
NM_007807	13058	Cybb	2.04651283	-0.2443704	4.35E-05	0.00163906
NM_010510	15977	Ifnb1	2.05146572	0.58111038	1.25E-08	9.99E-07
NM_178365	23969	Pacsin1	2.05321697	-0.8358555	0.00111467	0.02546119
NM_175648	244183	Trim30b	2.09466065	-1.0084944	0.00180455	0.03743389
NM_001164327	236451	Gm4902	2.16844422	4.78912877	8.40E-64	5.97E-60
NM_011579	21822	Tgtp1	2.18822096	2.69885592	1.65E-32	2.35E-29
NM_001024230	432555	Gm5431	2.19783884	2.23242211	5.00E-25	3.75E-22
NM_010050	13371	Dio2	2.2361448	-0.920153	0.00078	0.01900641
NM_013927	30952	Cngb3	2.32394488	2.0667889	1.48E-23	8.94E-21
NM_175678	319239	Npsr1	2.36779033	-0.8346889	0.00023674	0.0071915
NM_010821	17476	Mpeg1	2.55325025	5.29187466	3.05E-89	8.69E-85
NM_011832	23920	Insrr	2.59662077	-0.6789766	1.60E-05	0.00067256
NM_175026	236312	Pyhin1	2.74722	1.4525803	4.82E-22	2.59E-19
NM_010266	14544	Gda	2.75751772	2.74867866	1.80E-49	5.69E-46
NR_004051	81497	Btnl5	2.87546004	-1.4246425	0.00260597	0.04925541
NM_028967	74481	Batf2	2.8972481	-0.0574743	9.87E-09	8.17E-07
NM_008505	16909	Lmo2	3.08714552	-0.3183888	6.51E-08	4.59E-06
NM_001142337	16909	Lmo2	3.08714576	-0.3183888	6.52E-08	4.59E-06
NM_001142336	16909	Lmo2	3.08714592	-0.3183888	6.52E-08	4.59E-06
NM_001142335	16909	Lmo2	3.08714617	-0.3183888	6.52E-08	4.59E-06
NM_008782	18507	Pax5	3.49510524	-1.6186587	0.00199784	0.04044141
NM_001198835	12810	Coch	3.60842832	-1.5506963	0.00108339	0.02494006
NM_007728	12810	Coch	3.60842858	-1.5506963	0.00108397	0.02494006
NM_001037865	213945	Col28a1	5.47370385	-1.8402451	0.00116518	0.02636109
