# Supplementary material for: Inhibition of Hedgehog-Signaling Driven Genes in Prostate Cancer Cells by Sutherlandia frutescens Extract
Source: PLoS One. 2015 Dec 28;10(12):e0145507. doi: 10.1371/journal.pone.0145507 (PMC4694108; doi:10.1371/journal.pone.0145507)
Supplement: S2 Table — (PDF) [file pone.0145507.s003.pdf]

Supplement Table 2. Hh responsive genes

GenBank Nucleotide Accession	Gene ID	symbol	logFC	logCPM	PValue	FDR
NM_001136057	12891	Cpne6	-3.6395236	-0.9019861	8.31E-05	0.00420088
NR_001460	19782	Rmrp	-3.5213144	1.99792897	8.61E-06	0.0006202
NM_025427	66214	1190002H23Rik	-2.9539128	-1.3554094	0.00097882	0.03112639
NM_173749	210622	Pamr1	-2.8402228	-0.2595863	7.06E-08	8.63E-06
NR_002142	85029	Rpph1	-2.7728779	1.33112035	0.00041687	0.01627505
NM_130858	104079	Nxph3	-2.5829369	-1.2162615	0.00112165	0.03464668
NM_001205036	100504608	LOC100504608	-2.2419904	-0.6850822	0.00016592	0.00755579
NM_008607	17386	Mmp13	-1.9713698	5.05741074	3.48E-61	1.98E-57
NM_010266	14544	Gda	-1.7390841	0.31301462	4.35E-06	0.00033802
NM_009264	20753	Sprr1a	-1.7374851	2.27703935	1.70E-11	3.58E-09
NM_008964	19217	Ptger2	-1.6215138	-0.2829216	0.00067128	0.02340986
NM_177776	276829	Smtnl2	-1.5048487	2.05810539	2.22E-06	0.00019172
NM_029415	75750	Slc10a6	-1.4957894	1.24532274	3.14E-08	4.04E-06
NM_173027	271424	Ip6k3	-1.4853409	0.2030895	0.00013036	0.00616291
NM_008630	17750	Mt2	-1.3931301	4.27689067	2.11E-16	7.69E-14
NM_001199151	67847	Sncaip	-1.3824463	1.97601232	1.22E-11	2.60E-09
NM_001100462	434325	Tmem221	-1.3771544	-0.0183907	0.00163506	0.04658212
NM_010234	14281	Fos	-1.3332778	2.96296348	5.11E-16	1.80E-13
NM_001081416	68655	Fndc1	-1.286522	3.58858981	3.09E-20	1.73E-17
NM_009062	19736	Rgs4	-1.2662518	1.17611208	1.82E-06	0.00016176
NM_010889	17996	Neb	-1.2382907	0.45686183	0.00038383	0.01540193
NM_001033263	216439	Agap2	-1.2371415	3.35941217	1.11E-19	5.83E-17
NM_011414	20568	Slpi	-1.1692445	0.59338976	0.00045124	0.01737853
NM_010809	17392	Mmp3	-1.1446762	6.51858136	3.58E-40	4.85E-37
NM_029116	74901	Kbtbd11	-1.0828285	2.01670344	1.25E-06	0.00011629
NM_001159562	16181	Il1rn	-1.0697606	2.37949655	2.14E-08	2.79E-06
NM_009285	20855	Stc1	-1.0519635	5.38576534	6.64E-31	7.00E-28
NM_008909	19041	Ppl	-1.0477792	3.09133416	5.57E-13	1.43E-10
NM_013602	17748	Mt1	-1.0356728	5.57451377	7.92E-26	6.44E-23
NM_025961	67092	Gatm	-1.0130343	3.98117679	1.46E-14	4.30E-12
NM_008397	16403	Itga6	1.02682904	3.53933087	1.86E-15	6.17E-13
NM_030889	81840	Sorcs2	1.03194061	4.7520699	4.40E-20	2.41E-17
NM_001042611	12870	Cp	1.03206485	4.75911873	3.10E-22	2.16E-19
NM_207663	233335	Synm	1.0369468	1.58507736	6.34E-06	0.0004702
NM_194060	329934	Foxo6	1.0461692	1.81975346	9.92E-07	9.38E-05
NM_144800	211401	Mtss1	1.07478739	5.04547364	1.88E-28	1.78E-25
NM_008055	14366	Fzd4	1.0984462	2.18983872	1.65E-07	1.84E-05
NM_008506	16918	Mycl1	1.09878931	2.11726983	1.13E-08	1.59E-06
NM_011518	20963	Sykb	1.10933043	1.89537613	1.01E-07	1.19E-05
NM_001113379	434215	Lrrc32	1.14047899	6.31637179	1.30E-30	1.32E-27
NM_019427	54357	Epb4.1l4b	1.14101499	1.59948777	1.18E-06	0.00010981
NM_028903	71145	Scara5	1.14997839	5.71538084	4.68E-38	5.33E-35
NM_172285	234779	Plcg2	1.16567542	0.69508538	0.0002138	0.00937582
NM_016719	50915	Grb14	1.18509949	2.95026825	1.29E-12	3.16E-10
NM_008741	18197	Nsg2	1.22498959	0.86523233	7.63E-05	0.00394656
NM_001083895	68792	Srpx2	1.22583255	2.9514201	1.20E-12	2.96E-10
NM_172439	170835	Inpp5j	1.23160905	0.30172374	0.00084538	0.02764256
NM_133721	104099	Itga9	1.23704574	1.23758506	1.70E-06	0.00015259
NM_007805	13056	Cyb561	1.24193194	2.48221279	9.50E-13	2.41E-10
NM_001033548	436022	6030429G01Rik	1.2471597	0.36591515	0.00054728	0.02033051
NM_001109657	14457	Gas7	1.26495869	5.46177396	4.75E-40	5.88E-37

NM_001161665	269152	Kif26b	1.2712679	0.95715014	1.88E-05	0.00123594
NM_008882	18845	Plxna2	1.29043296	4.13635878	4.32E-28	3.85E-25
NM_018805	54710	Hs3st3b1	1.29048258	1.75088905	4.24E-09	6.52E-07
NM_009846	12484	Cd24a	1.2910287	7.16334717	9.40E-54	2.06E-50
NM_008580	26408	Map3k5	1.29765796	0.78350064	2.34E-05	0.00148985
NM_001103177	226251	Ablim1	1.30681413	7.12869963	8.40E-59	3.00E-55
NM_001111314	53972	Ngef	1.3204835	2.32712458	1.34E-12	3.24E-10
NM_013454	11303	Abca1	1.32745232	3.98019328	8.40E-28	7.25E-25
NM_028829	74229	Paqr8	1.35665356	4.37652119	1.11E-35	1.21E-32
NM_148933	108115	Slco4a1	1.35823711	0.83379317	1.36E-05	0.00092702
NM_134117	106522	Pkdcc	1.36417353	6.78132671	2.87E-61	1.98E-57
NM_001081052	195727	Nhs	1.39933888	1.86607927	8.95E-11	1.72E-08
NM_026346	67731	Fbxo32	1.40277191	6.09637571	1.18E-57	3.73E-54
NM_198673	382075	Odf3l1	1.41886464	-0.1211422	0.00097192	0.03094164
NM_007539	12061	Bdkrb1	1.42189009	-0.1116534	0.00109095	0.03393401
NM_001001488	54670	Atp8b1	1.42472001	-0.1119119	0.00120778	0.03676439
NM_001003948	98496	Pid1	1.44305993	3.61978909	2.05E-28	1.88E-25
NM_001170847	73713	Rbm20	1.46431428	1.08882897	2.60E-07	2.75E-05
NM_201352	233552	Gdpd5	1.48884184	1.24733117	1.48E-08	2.04E-06
NM_009641	11602	Angpt4	1.49340635	0.52564867	1.67E-05	0.00111272
NM_007930	13803	Enc1	1.49764848	7.05847447	1.46E-72	1.38E-68
NM_026878	68939	Rasl11b	1.547206	2.94823264	1.32E-23	1.02E-20
NM_028804	74186	Ccdc3	1.58760216	2.80665019	6.07E-15	1.88E-12
NM_030725	80976	Syt13	1.67951995	0.8273998	1.10E-07	1.28E-05
NM_007431	11647	Alpl	1.69001924	1.71916725	1.50E-12	3.56E-10
NM_010181	14119	Fbn2	1.70680325	0.69247524	3.38E-06	0.00027341
NM_008235	15205	Hes1	1.77226604	2.37689251	1.58E-19	8.04E-17
NM_022032	64058	Perp	1.77763535	0.72872132	4.43E-08	5.60E-06
NM_028472	73230	Bmper	1.79965779	3.85621879	3.47E-42	5.20E-39
NM_022315	64074	Smoc2	1.81986242	5.25802847	5.64E-57	1.60E-53
NM_008813	18605	Enpp1	1.82227017	3.60862496	1.12E-38	1.32E-35
NM_011641	22061	Trp63	1.86104867	1.82464722	6.72E-17	2.50E-14
NM_008242	15229	Foxd1	1.91649505	0.89468737	5.11E-10	9.03E-08
NM_021399	58208	Bcl11b	1.91777603	0.22423161	1.27E-06	0.00011694
NM_205823	384059	Tlr12	1.95761349	-0.3835123	0.00011297	0.00542179
NM_080467	140494	Atp6v0a4	2.00090756	0.83660961	3.20E-10	5.88E-08
NM_207624	11421	Ace	2.05111688	1.6325882	1.43E-15	4.77E-13
NM_001081171	16776	Lama5	2.06503871	3.82977573	3.22E-56	7.64E-53
NM_001002927	18619	Penk	2.09633225	4.17175706	2.76E-52	5.61E-49
NM_009928	12819	Col15a1	2.11404664	3.7780145	1.31E-56	3.39E-53
NM_008957	19206	Ptch1	2.1322943	5.16000812	7.34E-42	1.05E-38
NM_022316	64075	Smoc1	2.14698155	4.09351634	3.35E-52	5.96E-49
NM_008289	15484	Hsd11b2	2.19342168	-0.9047726	0.00070688	0.0242678
NM_020293	56863	Cldn9	2.26542041	-0.4807985	2.50E-05	0.00157485
NM_153393	237759	Col23a1	2.27177951	3.57851309	1.63E-51	2.73E-48
NM_001039554	654812	Angptl7	2.37352853	-1.0308408	0.00066392	0.02324199
NM_010518	16011	Igfbp5	2.39858061	4.76819892	7.16E-92	1.02E-87
NM_026496	252973	Grhl2	2.4724145	-1.2652445	0.00175555	0.04912938
NM_001039347	56543	Kcnd3	2.65395746	0.24879013	4.11E-10	7.32E-08
NR_033261	677289	Gm14492	2.6609846	0.60254209	5.55E-12	1.23E-09
NM_028799	74176	Tgm5	2.75388137	0.04897287	1.73E-09	2.88E-07
NM_001163145	72301	1810041L15Rik	2.86104242	-0.7007932	5.53E-06	0.00041742
NM_172791	237625	Pla2g3	2.88314193	-0.9942494	6.51E-05	0.00345757

NM_030143	73284	Ddit4l	3.42466846	1.12760611	3.76E-24	2.98E-21
NM_001044751	15483	Hsd11b1	3.57350559	0.55053924	2.77E-17	1.11E-14
NM_009747	12062	Bdkrb2	3.70755472	2.2702199	3.70E-50	5.85E-47
NM_008958	19207	Ptch2	4.00712709	-1.2545657	2.98E-05	0.0018237
NM_010296	14632	Gli1	5.26070944	4.37251754	9.12E-206	2.60E-201
NM_020263	56808	Cacna2d2	6.33390639	-1.3078387	2.65E-06	0.00022297
