# Supplementary material for: Inhibition of Hedgehog-Signaling Driven Genes in Prostate Cancer Cells by Sutherlandia frutescens Extract
Source: PLoS One. 2015 Dec 28;10(12):e0145507. doi: 10.1371/journal.pone.0145507 (PMC4694108; doi:10.1371/journal.pone.0145507)
Supplement: S3 Table — (PDF) [file pone.0145507.s004.pdf]

Supplement Table 3. Hh+Sutherlandia extract 80ug/ml vs Hh

GenBank Nucleotide Accession	Gene ID	symbol	logFC	logCPM	PValue	FDR
NM_020263	56808	Cacna2d2	-6.2250421	-1.236515	4.21E-06	9.35E-05
NM_173394	225471	Ticam2	-5.243686	-1.8688164	0.00400376	0.0401993
NM_021397	58206	Zbtb32	-5.2432537	-1.8722146	0.00400425	0.0401993
NM_170593	214240	Disp2	-5.2426388	-1.8756081	0.00403335	0.0403917
NM_030143	73284	Ddit4l	-3.4585717	1.14366194	2.63E-23	2.55E-21
NM_172655	329152	Hecw2	-3.1874601	-1.5880507	0.00356551	0.03662139
NM_001039347	56543	Kcnd3	-2.7431012	0.26896407	1.70E-10	7.03E-09
NM_015732	12006	Axin2	-2.6432055	-0.6195087	4.50E-05	0.00083079
NM_029771	76854	Gpr30	-2.6424976	-0.1671999	8.41E-07	2.16E-05
NM_010181	14119	Fbn2	-2.3501505	0.595241	4.40E-09	1.53E-07
NM_018805	54710	Hs3st3b1	-2.3221786	1.53528974	1.91E-19	1.43E-17
NM_022316	64075	Smoc1	-2.3116568	4.05036947	3.62E-92	2.78E-89
NM_008289	15484	Hsd11b2	-2.277234	-0.8723441	0.0009869	0.01247808
NM_172791	237625	Pla2g3	-2.2763174	-0.8807985	0.00159495	0.01868824
NM_010296	14632	Gli1	-2.1961272	4.60453248	1.38E-101	1.46E-98
NM_016851	54139	Irf6	-2.1900116	-0.6921811	0.00057815	0.00792616
NM_020333	57138	Slc12a5	-2.0828964	-0.563565	0.00033914	0.0050141
NM_001160163	23956	Neu2	-1.9884004	-0.6217735	0.00089149	0.01145489
NM_010518	16011	Igfbp5	-1.9035572	4.84436753	8.25E-79	4.70E-76
NM_011641	22061	Trp63	-1.8781197	1.82474367	3.38E-17	2.21E-15
NM_009747	12062	Bdkrb2	-1.8071668	2.51695768	1.73E-23	1.69E-21
NM_198673	382075	Odf3l1	-1.7683807	-0.1737649	0.00017459	0.00280731
NM_022032	64058	Perp	-1.7428701	0.7512754	1.50E-07	4.30E-06
NM_013599	17395	Mmp9	-1.7113001	0.79019419	1.01E-07	2.94E-06
NM_212487	332131	Krt78	-1.6961795	-0.2186425	0.00038052	0.00555377
NM_001163145	72301	1810041L15Rik	-1.681231	-0.4990189	0.00177951	0.02043032
NR_033261	677289	Gm14492	-1.6812248	0.78194413	2.72E-07	7.54E-06
NM_008242	15229	Foxd1	-1.5793486	0.97366555	1.37E-07	3.93E-06
NM_008957	19206	Ptch1	-1.5704079	5.26584175	8.68E-68	3.80E-65
NM_027724	71213	Cage1	-1.5602183	-0.4399132	0.00372422	0.03799105
NM_001172160	71436	Flrt3	-1.4231312	0.81634932	4.96E-06	0.00010885
NR_045182	73326	4932702P03Rik	-1.4130187	-0.4081356	0.0047272	0.04609138
NM_009928	12819	Col15a1	-1.4105971	3.92206181	2.10E-32	2.91E-30
NM_030725	80976	Syt13	-1.4005716	0.90038983	2.37E-06	5.52E-05
NM_080467	140494	Atp6v0a4	-1.3958744	0.97707622	1.31E-06	3.18E-05
NM_019867	53972	Ngef	-1.371738	2.32237497	8.08E-14	4.30E-12
NM_001024954	80720	Pbx4	-1.3660071	-0.3114674	0.0039908	0.04012083
NM_001190913	67703	Kirrel3	-1.3539503	0.34977873	0.00031203	0.00466911
NM_026346	67731	Fbxo32	-1.3429889	6.09582956	1.49E-96	1.28E-93
NM_008966	19220	Ptgfr	-1.3364208	3.23310201	6.96E-18	4.77E-16
NM_205823	384059	Tlr12	-1.3264419	-0.2325589	0.00366675	0.0374585
NM_028973	74488	Lrrc15	-1.3226501	1.24967157	3.02E-07	8.29E-06
NM_053244	114229	Kiss1r	-1.3083694	0.50625628	0.00023746	0.00368105
NM_153393	237759	Col23a1	-1.2755032	3.79201058	3.15E-21	2.66E-19
NM_021877	15460	Hr	-1.2672452	0.07971858	0.00203492	0.02290071
NM_007930	13803	Enc1	-1.251723	7.10886007	9.61E-113	1.14E-109
NM_013464	11622	Ahr	-1.2506491	5.33891401	1.54E-56	4.62E-54
NM_028977	74511	Lrrc17	-1.2417166	0.13576926	0.00246078	0.02689564
NM_008741	18197	Nsg2	-1.2180259	0.86920691	4.16E-05	0.00077637
NM_001002927	18619	Penk	-1.2119043	4.37089639	1.42E-39	2.52E-37
NM_001103178	226251	Ablim1	-1.2099486	7.14737132	4.67E-83	3.09E-80

NM_007539	12061	Bdkrb1	-1.1968248	-0.042041	0.00480574	0.04658584
NM_028804	74186	Ccdc3	-1.1806917	2.90801121	6.10E-16	3.65E-14
NM_207280	403180	Ccdc121	-1.1645376	0.82801591	0.00010343	0.00175856
NM_201531	382571	Kcnf1	-1.1565199	0.63289092	0.00040175	0.00583683
NM_009871	12569	Cdk5r1	-1.1493867	0.58371388	0.00070139	0.00933685
NM_028472	73230	Bmper	-1.1341897	4.01665418	2.86E-25	2.96E-23
NM_001081171	16776	Lama5	-1.1179089	4.05104105	1.10E-27	1.22E-25
NM_001044751	15483	Hsd11b1	-1.1059729	0.9581144	0.00011693	0.00196951
NM_207624	11421	Ace	-1.1011094	1.86079886	7.14E-08	2.12E-06
NM_172803	238130	Dock4	-1.0828102	0.42891141	0.00162603	0.01902398
NM_021399	58208	Bcl11b	-1.0825945	0.43210828	0.00262821	0.0283845
NM_026878	68939	Rasl11b	-1.0825198	3.06964363	8.55E-15	4.85E-13
NM_027921	71781	Slc16a14	-1.0821372	0.30108261	0.00331343	0.03432969
NM_028247	72472	Slc16a10	-1.072972	0.94190531	0.00023463	0.00364716
NM_001161483	319211	Nol4	-1.0696354	0.35467305	0.00293576	0.03102665
NM_172439	170835	Inpp5j	-1.0693314	0.35859321	0.00264621	0.02856042
NM_194060	329934	Foxo6	-1.0610282	1.81387937	6.02E-07	1.58E-05
NM_001170847	73713	Rbm20	-1.0582198	1.20206523	5.39E-05	0.00097891
NR_040560	100038453	Gm12522	-1.0569125	0.40712858	0.00473028	0.04610562
NM_028829	74229	Paqr8	-1.0287545	4.46085827	8.94E-29	1.04E-26
NM_001033548	436022	6030429G01Rik	-1.0214109	0.43815257	0.00349579	0.03603123
NM_015755	26559	Hunk	-1.0197575	1.53033913	1.24E-05	0.00025525
NM_018779	54611	Pde3a	-1.0126728	1.69830282	4.02E-06	8.98E-05
NM_001170401	268567	Tmem229b	1.00111812	3.25446671	2.03E-15	1.17E-13
NM_013484	12263	C2	1.00312468	0.99133494	0.00113997	0.01419893
NM_001034859	225594	Gm4841	1.00790851	5.32694576	4.85E-45	9.72E-43
NM_023141	30935	Tor3a	1.00832276	8.89852005	3.36E-84	2.33E-81
NM_001113527	57444	Isg20	1.00837247	5.63727589	6.50E-51	1.54E-48
NM_019440	54396	Irgm2	1.01329997	8.36130604	4.07E-97	3.73E-94
NM_023738	74153	Uba7	1.01505187	4.75001493	2.74E-35	4.20E-33
NM_008479	16768	Lag3	1.01716293	1.81564947	1.11E-06	2.73E-05
NM_009399	21934	Tnfrsf11a	1.02005606	1.4843369	1.13E-05	0.00023366
NM_009627	11535	Adm	1.02438138	3.31073936	6.05E-16	3.63E-14
NM_008357	16168	Il15	1.03646124	1.8063205	4.32E-07	1.16E-05
NM_178779	320311	Rnf152	1.04563173	0.57540268	0.00146221	0.01745784
NM_025992	67138	Herc6	1.04959935	6.58230401	1.00E-65	3.66E-63
NM_199016	224794	Enpp4	1.05160557	5.69480813	1.60E-54	4.26E-52
NM_013566	16421	Itgb7	1.05179283	1.71101488	8.95E-07	2.28E-05
NM_011243	218772	Rarb	1.05568349	1.29113023	1.64E-05	0.00032859
NM_175118	67446	Dusp28	1.05583455	2.37722542	6.42E-09	2.17E-07
NM_001171007	107607	Nod1	1.05627313	5.09242952	3.70E-36	5.92E-34
NM_134102	85031	Pla1a	1.05651633	3.3595494	3.34E-18	2.32E-16
NM_001101605	667373	Gm14446	1.05693703	2.26615652	2.77E-09	9.91E-08
NM_011414	20568	Slpi	1.05746915	0.49341647	0.00275726	0.02955715
NM_175164	71302	Arhgap26	1.05878613	0.50407585	0.00154401	0.01817423
NR_027886	68074	A930013F10Rik	1.06055745	0.3046823	0.00344813	0.03555692
NM_001038643	108116	Slco3a1	1.06139263	2.61893537	6.14E-12	2.86E-10
NM_007568	12223	Btc	1.06160919	3.82719553	1.11E-22	1.04E-20
NM_001001892	14972	H2-K1	1.07644137	8.97880168	5.33E-86	3.79E-83
NM_172777	236573	Gbp9	1.07656648	5.78266559	1.70E-49	3.88E-47
NM_011852	23960	Oas1g	1.07687856	5.24399585	4.93E-35	7.51E-33
NM_183201	327978	Slfn5	1.08019897	1.50889896	3.84E-06	8.61E-05
NM_011610	21938	Tnfrsf1b	1.08146822	5.71467542	2.34E-56	6.86E-54

NM_001033196	98999	Znfx1	1.08185502	8.2132178	2.38E-103	2.60E-100
NM_001159393	16362	Irf1	1.09038039	5.11788571	1.30E-32	1.81E-30
NM_145227	246728	Oas2	1.09273619	6.70054055	9.72E-77	5.12E-74
NM_007592	12319	Car8	1.10044353	1.7772771	9.09E-08	2.66E-06
NM_011909	24110	Usp18	1.1026372	7.42656299	4.81E-66	1.83E-63
NM_020581	57875	Angptl4	1.11081455	1.13959069	2.94E-05	0.00056313
NM_009640	11600	Angpt1	1.11139034	4.45922614	7.05E-35	1.07E-32
NM_145515	226778	Mark1	1.11145139	4.4185387	1.59E-31	2.13E-29
NM_029035	74646	Spsb1	1.11955691	3.89019643	6.81E-25	6.94E-23
NM_001111267	211329	Ncoa7	1.12974781	4.20922539	7.09E-18	4.84E-16
NM_001127330	19016	Pparg	1.13172226	1.89759834	5.27E-07	1.39E-05
NM_001164627	73167	Arhgap8	1.13221236	1.95159225	3.97E-08	1.22E-06
NM_008791	18546	Pcp4	1.1330954	0.14003911	0.00392762	0.03968194
NM_001039701	16181	Il1rn	1.13329074	2.41315533	1.15E-11	5.28E-10
NM_021443	20307	Ccl8	1.14723576	0.31285039	0.0014575	0.01742204
NM_010872	17948	Naip2	1.15037242	1.43411256	3.07E-06	7.02E-05
NM_009890	12642	Ch25h	1.15162407	3.93335303	6.13E-28	6.87E-26
NM_013585	16912	Psmb9	1.16127088	4.36657411	2.00E-35	3.08E-33
NM_011454	20708	Serpinb6b	1.16447097	3.93358779	1.54E-28	1.78E-26
NM_013834	20377	Sfrp1	1.16934463	6.21381255	1.03E-81	6.39E-79
NM_001081047	194231	Cnksr1	1.16989142	0.22546116	0.00253207	0.02755843
NM_028351	72780	Rspo3	1.1716521	5.23098317	2.81E-56	8.16E-54
NM_010597	16497	Kcnab1	1.17992586	2.12421778	1.31E-09	4.86E-08
NM_033622	24099	Tnfsf13b	1.18301995	3.37050356	7.83E-21	6.48E-19
NM_013606	17858	Mx2	1.18979186	6.03753734	8.30E-52	2.04E-49
NM_008230	15186	Hdc	1.19903911	1.72807491	1.76E-08	5.65E-07
NM_001143689	15015	H2-Q4	1.20116956	6.51978446	7.03E-66	2.63E-63
NR_004446	630499	H2-K2	1.20153805	2.37949115	3.62E-11	1.59E-09
NM_008328	15950	Ifi203	1.20288822	7.82079125	2.62E-82	1.70E-79
NM_029653	69635	Dapk1	1.20480596	0.30178926	0.00140791	0.01695033
NM_139198	231507	Plac8	1.20551097	4.15119659	4.17E-23	4.00E-21
NM_199015	219132	D14Ertd668e	1.2063072	6.65385976	8.53E-81	5.16E-78
NM_181390	66175	Mustn1	1.21263762	2.79529555	2.74E-16	1.66E-14
NM_012057	27056	Irf5	1.21364061	4.96844519	3.17E-53	8.21E-51
NM_027828	104943	Fam110c	1.2143616	2.31248529	2.82E-12	1.35E-10
NM_126166	142980	Tlr3	1.21474247	7.34817828	2.18E-114	2.82E-111
NM_001136059	13076	Cyp1a1	1.22003273	0.95794061	1.97E-05	0.00038726
NM_001164289	628705	Gm6907	1.22169308	1.06084889	8.72E-06	0.00018311
NM_001081024	239122	Setdb2	1.22487263	5.33471991	3.86E-52	9.64E-50
NM_018866	55985	Cxcl13	1.23228544	-0.124261	0.00377363	0.03833963
NM_010927	18126	Nos2	1.23310716	2.02283601	1.89E-10	7.77E-09
NM_016850	54123	Irf7	1.23564677	7.85906383	1.92E-125	3.04E-122
NM_001005858	667370	I830012O16Rik	1.23808231	8.2610984	2.08E-76	1.07E-73
NM_172812	15558	Htr2a	1.24116078	3.08810306	1.19E-17	8.00E-16
NM_010501	15959	Ifit3	1.24313875	9.92386907	5.09E-77	2.73E-74
NM_007865	13388	Dll1	1.24515418	1.76514144	8.55E-09	2.83E-07
NM_013655	20315	Cxcl12	1.24892705	8.90358021	1.86E-97	1.76E-94
NM_011193	19200	Pstpip1	1.25128627	1.87323625	1.60E-09	5.90E-08
NM_007797	13025	Ctla2b	1.26569532	0.27169672	0.00070832	0.00942039
NM_001045540	620913	Gm12185	1.2669615	2.87108843	1.95E-18	1.38E-16
NM_025829	66892	Eif4e3	1.27569457	0.41246208	0.00166325	0.01935317
NM_033616	114564	Csprs	1.28563163	5.28283767	1.98E-62	6.64E-60
NM_197944	209488	Hsh2d	1.29753209	1.91228098	1.21E-10	5.05E-09

NM_148927	69217	Plekha4	1.29942925	3.11700392	1.97E-20	1.59E-18
NM_008161	14778	Gpx3	1.30085707	2.78570941	9.19E-17	5.77E-15
NM_007695	12654	Chi3l1	1.30715875	0.65717201	5.05E-05	0.00092236
NM_001039223	623781	Gm14137	1.31046847	2.19693194	1.99E-12	9.61E-11
NM_175930	217944	Rapgef5	1.31422286	-0.0858933	0.00248421	0.02712045
NM_011408	20556	Slfn2	1.33028128	6.2631857	1.30E-72	6.40E-70
NM_001243837	109828	C7	1.33138946	0.99243552	3.02E-06	6.92E-05
NM_010720	16891	Lipg	1.33165147	3.26355677	1.09E-14	6.13E-13
NM_008343	16009	Igfbp3	1.33412171	6.17460087	7.05E-87	5.14E-84
NM_020557	22169	Cmpk2	1.33974469	8.10791427	1.92E-136	3.91E-133
NM_001139519	58203	Zbp1	1.35140514	5.27750285	1.24E-67	5.27E-65
NM_010260	14469	Gbp2	1.35434609	8.33608385	2.20E-110	2.51E-107
NM_007446	11722	Amy1	1.35776219	-0.1325044	0.0027851	0.02979951
NM_023124	15019	H2-Q8	1.35922091	-0.1263865	0.00191179	0.02170382
NM_008727	18160	Npr1	1.3592717	0.27996712	0.00018408	0.00294006
NM_008381	16324	Inhbb	1.37027654	0.73411938	1.31E-05	0.0002677
NM_007986	14089	Fap	1.37719147	1.3998524	6.97E-09	2.34E-07
NM_011723	22436	Xdh	1.38226289	8.05114012	1.61E-126	2.69E-123
NM_018738	16145	Igtp	1.40634985	8.28194665	1.44E-127	2.73E-124
NM_008404	16414	Itgb2	1.40686168	1.38920065	5.28E-09	1.81E-07
NM_011820	23887	Ggt5	1.44888121	2.07467958	9.19E-13	4.55E-11
NM_008880	18828	Plscr2	1.45714626	5.50964282	9.27E-73	4.63E-70
NM_001013817	434484	Sp140	1.46678753	3.33642256	8.25E-30	1.01E-27
NM_010279	14585	Gfra1	1.47116024	4.32739579	1.08E-46	2.27E-44
NM_178098	214639	4930486L24Rik	1.4775898	-0.0504321	0.00042525	0.0061188
NM_001142706	14962	Cfb	1.48794123	2.23311327	2.60E-16	1.58E-14
NM_001081746	665378	Gm7609	1.48980853	2.93717845	3.12E-21	2.64E-19
NM_009062	19736	Rgs4	1.49320433	1.30088821	6.34E-09	2.15E-07
NM_001163621	71939	Apol6	1.5094348	3.27896158	6.34E-26	6.68E-24
NM_011474	20762	Sprr2h	1.51034881	-0.2129757	0.00352034	0.03622282
NM_008332	15958	Ifit2	1.52061289	8.94090712	8.87E-143	1.94E-139
NM_008330	15953	Ifi47	1.53076978	6.53511993	4.20E-125	6.30E-122
NM_001033207	434341	Nlrc5	1.53180369	5.5608354	1.30E-90	9.77E-88
NM_173767	233752	Insc	1.53584468	0.82667479	6.05E-06	0.00013084
NM_008599	17329	Cxcl9	1.53735573	2.19510378	6.37E-16	3.80E-14
NM_029415	75750	Slc10a6	1.54664519	1.24896561	6.43E-08	1.93E-06
NM_153564	229898	Gbp5	1.55134721	6.29030581	1.77E-127	3.14E-124
NR_003967	209380	Gm4759	1.55862661	0.91102218	6.39E-08	1.92E-06
NM_007796	13024	Ctla2a	1.56629133	1.90123744	4.53E-14	2.45E-12
NM_007870	13421	Dnase1l3	1.56821958	-0.0733626	0.00019468	0.00308166
NM_001039160	74558	Gvin1	1.57648932	3.61115929	3.27E-31	4.25E-29
NM_001243039	100042856	Gm4070	1.57649091	3.61115929	3.24E-31	4.23E-29
NM_021893	60533	Cd274	1.60040554	4.91441514	6.41E-71	3.04E-68
NM_008353	16161	Il12rb1	1.63164631	1.08715693	4.59E-09	1.59E-07
NM_001114679	667214	9930111J21Rik1	1.65601571	4.25728302	3.11E-65	1.09E-62
NM_010426	15227	Foxf1a	1.67146261	1.6878496	2.40E-14	1.32E-12
NM_001168504	232889	Pla2g4c	1.70349725	0.24641122	1.08E-05	0.00022325
NM_175397	109032	Sp110	1.71393021	4.15902722	9.59E-60	3.03E-57
NM_172603	219131	Phf11	1.7213381	2.97898226	1.01E-26	1.10E-24
NM_001045543	629303	Gm11435	1.72621826	0.18734051	1.18E-05	0.00024321
NM_001135115	631323	Gm12250	1.7470523	5.97000259	5.82E-150	1.50E-146
NM_021384	58185	Rsad2	1.76164404	9.35097492	1.34E-223	1.27E-219
NM_177981	15114	Hap1	1.77250246	3.91960877	1.31E-58	4.09E-56

NM_009264	20753	Sprr1a	1.77649263	2.2859325	6.95E-22	6.22E-20
NM_008620	17472	Gbp4	1.78926568	3.92575274	3.23E-61	1.04E-58
NM_011226	19331	Rab19	1.80766899	-0.0888624	6.03E-05	0.00107754
NM_009780	12268	C4b	1.83814608	0.72842028	1.47E-08	4.74E-07
NM_011704	22361	Vnn1	1.8415045	4.38870809	7.83E-84	5.31E-81
NM_021274	15945	Cxcl10	1.84178327	8.55916716	1.56E-192	6.36E-189
NR_040347	328576	A430075N02	1.84843061	-0.3940711	0.0003262	0.0048481
NM_009763	12182	Bst1	1.85657617	-0.7962999	0.0015206	0.01797256
NM_011303	20148	Dhrs3	1.87001952	4.98120064	3.34E-115	4.53E-112
NM_145209	231655	Oasl1	1.89214011	6.00410126	1.46E-167	4.62E-164
NM_001039646	626578	Gbp10	1.91899623	2.78893781	7.22E-35	1.09E-32
NM_010680	16774	Lama3	1.92143752	0.32798069	3.09E-07	8.48E-06
NM_001145164	100039796	Tgtp2	1.92636117	5.21998947	4.07E-101	4.14E-98
NM_011979	26464	Vnn3	1.93848475	2.76164407	1.16E-32	1.61E-30
NM_017466	54199	Ccrl2	1.9662825	2.8727129	3.73E-36	5.93E-34
NM_010999	18356	Olfr56	1.97106792	0.11140383	1.42E-06	3.44E-05
NM_010531	16068	Il18bp	1.97907599	3.27728774	2.07E-45	4.20E-43
NM_029509	76074	Gbp8	1.98135179	-0.7157381	0.00065548	0.00878732
NM_001100462	434325	Tmem221	1.99820141	0.37679488	3.16E-08	9.86E-07
NM_031168	16193	Il6	2.00188198	1.00918197	1.47E-11	6.67E-10
NR_030719	668108	Gm8979	2.0035619	-0.880121	0.00159299	0.01867298
NM_001037925	625360	BC147527	2.0156868	-0.5525088	0.00022685	0.00353963
NM_025658	66607	Ms4a4d	2.03146724	4.45118032	2.52E-98	2.47E-95
NM_030701	80885	Niacr1	2.03428551	-1.0602899	0.00277077	0.02966848
NM_001093775	17933	Myt1l	2.03522719	-1.054672	0.00283773	0.03022527
NM_001162955	71223	Gpr15	2.03537731	-1.0671845	0.00298315	0.03141832
NM_145949	209176	Ido2	2.03585881	-1.0576588	0.00286074	0.03043163
NM_001164329	628693	Gm6904	2.07863105	-1.2631345	0.00467337	0.04567401
NM_009425	22035	Tnfsf10	2.1100997	5.55063814	6.26E-147	1.49E-143
NM_010510	15977	Ifnb1	2.12384604	0.54936249	2.81E-09	1.00E-07
NR_003520	17857	Mx1	2.12929469	6.81910156	4.79E-222	2.72E-218
NM_001004174	433470	AA467197	2.13068984	1.20099583	1.31E-14	7.31E-13
NM_033073	110310	Krt7	2.13401848	-0.8026247	0.00034993	0.00515499
NM_001204910	226691	AI607873	2.15264125	6.24605483	2.27E-233	3.23E-229
NM_001033229	110115	Cyp11b1	2.17089361	-1.2139702	0.00294522	0.03110348
NM_023044	65221	Slc15a3	2.18446011	-0.9726274	0.00112497	0.01404289
NM_008392	16365	Irg1	2.18996588	2.49356971	2.69E-34	3.91E-32
NM_001033263	216439	Agap2	2.21368496	4.07804286	4.89E-94	3.98E-91
NM_028967	74481	Batf2	2.21716012	-0.0230732	3.23E-07	8.80E-06
NM_030218	78906	9130017N09Rik	2.22567506	1.53317809	1.51E-19	1.15E-17
NM_008029	14257	Flt4	2.25235803	-0.5437379	4.85E-05	0.0008892
NM_133211	170743	Tlr7	2.26072975	-1.4341215	0.00432756	0.04284062
NM_001039647	634650	Gbp11	2.2890245	2.41510668	2.60E-34	3.79E-32
NM_001162938	100033459	Pydc3	2.29920006	1.9766805	8.73E-28	9.74E-26
NM_008607	17386	Mmp13	2.31081023	5.31123383	3.47E-183	1.24E-179
NM_172648	226695	Ifi205	2.33125187	4.75644509	3.29E-123	4.68E-120
NM_007807	13058	Cybb	2.34644523	-0.4741955	1.66E-05	0.00033279
NM_001164683	382245	Tmem29	2.38178297	-0.838547	0.00024364	0.00376454
NM_175026	236312	Pyhin1	2.39373249	1.08966638	5.11E-16	3.09E-14
NM_011579	21822	Tgtp1	2.42586634	2.65602928	5.87E-46	1.20E-43
NM_001164327	236451	Gm4902	2.45820562	4.80793816	2.09E-153	5.94E-150
NM_011861	23969	Pacsin1	2.49007504	-0.2090702	4.60E-07	1.23E-05
NM_011832	23920	Insrr	2.50491245	-0.7524352	4.72E-05	0.00086834

NM_024237	70370	Fbln7	2.51511265	5.68646144	3.69E-222	2.62E-218
NM_013514	13829	Epb4.9	2.53433535	0.09858045	1.30E-08	4.22E-07
NM_013563	16186	Il2rg	2.55474284	-1.2497112	0.00271856	0.02920599
NM_001024230	432555	Gm5431	2.56533412	1.85167222	2.94E-29	3.48E-27
NM_001177351	107350	AW112010	2.60975228	0.38859776	2.38E-11	1.05E-09
NM_009888	12628	Cfh	2.61368941	1.89287322	4.74E-29	5.55E-27
NM_011141	18991	Pou3f1	2.65321522	0.05946758	7.74E-09	2.58E-07
NM_001168620	83965	Enpp5	2.68749969	-0.8712393	0.00019388	0.00307071
NM_008505	16909	Lmo2	2.76380839	-0.5733098	2.28E-06	5.32E-05
NM_001177349	623121	Pydc4	2.76877657	2.67347085	1.89E-51	4.60E-49
NM_025427	66214	1190002H23Rik	2.78641285	-1.4853407	0.00433116	0.04286127
NM_010050	13371	Dio2	2.79990141	-1.1087884	0.00015016	0.00246325
NM_145373	209588	Sectm1a	2.80641721	-0.7955645	1.77E-05	0.00035185
NM_019494	56066	Cxcl11	2.93968773	1.60482367	2.16E-29	2.59E-27
NM_145741	14560	Gdf10	3.01929844	-0.6446211	8.71E-07	2.23E-05
NM_010821	17476	Mpeg1	3.04552523	5.29322694	1.22E-249	3.46E-245
NM_001143686	328563	Apol11b	3.24949795	-1.2025972	4.27E-05	0.00079293
NM_013927	30952	Cngb3	3.2728443	1.95399738	2.75E-41	5.05E-39
NM_016767	53314	Batf	3.38764168	-1.6942528	0.00352372	0.03623262
NM_178703	232333	Slc6a1	3.51076923	-1.6220782	0.00189477	0.02151912
NR_045048	100503961	Gm19990	3.72942812	-1.4873646	0.00054517	0.00753939
NM_010266	14544	Gda	4.09835612	2.23953715	7.88E-64	2.67E-61
NM_011410	20558	Slfn4	4.23704449	-1.1411878	1.30E-05	0.00026683
NM_175678	319239	Npsr1	6.14793891	-1.4218292	1.62E-05	0.00032527
