# Supplementary material for: Inhibition of Hedgehog-Signaling Driven Genes in Prostate Cancer Cells by Sutherlandia frutescens Extract
Source: PLoS One. 2015 Dec 28;10(12):e0145507. doi: 10.1371/journal.pone.0145507 (PMC4694108; doi:10.1371/journal.pone.0145507)
Supplement: S4 Table — (PDF) [file pone.0145507.s005.pdf]

Supplement Table 4 Primer Sequences

GAPDH (NM_008084)	forward primer: AGCCTCGTCCCGTAGACAAAAT
	reverse primer: CCGTGAGTGGAGTCATACTGGA
Patched (NM_008957)	forward primer: CTCTGGAGCAGA TTTCCAAGG
	reverse primer: TGCCGCAGTTCTTTTGAATG
Gli1 (NM_010296)	forward primer: GGAAGTCCTATTCACGCCTTGA
	reverse primer: CAACCTTCTTGCTCACACATGTAAG
Hsd11b1 (NM_001044751)	forward primer: CTGCCTGCCTGGGAGGTTGT
	reverse primer: TCCCTGGAGCATTTCTGGTCTGAAC
